# Development and validation of the scale for measuring biopsychosocial approach of family physicians to their patients

**DOI:** 10.1136/fmch-2021-001407

**Published:** 2022-05-16

**Authors:** Irena Makivić, Zalika Klemenc-Ketiš

**Affiliations:** 1 National Institute of Public Health, Ljubljana, Slovenia; 2 Department of Family Medicine, University of Ljubljana Faculty of Medicine, Ljubljana, Slovenia; 3 Department of Family Medicine, Faculty of Medicine University of Maribor, Maribor, Slovenia

**Keywords:** Physicians, Family, Primary Health Care, Patient-Centered Care, Mental Health, Physician-Patient Relations

## Abstract

**Objective:**

While other models focus more on disease and pathophysiology, the biopsychosocial approach emphasises the importance of human health and disease in their fullest contexts. If we are to gain an insight into physical and psychological health needs, and address them quickly and adequately, it is important that we recognise them already at the family practice stage. An approach that assesses needs at patient level could also be seen as patient-centred care, which is one of the key elements of high-quality care. To the best of our knowledge, no scale for measuring the biopsychosocial approach of family physicians has yet been developed.

**Design:**

The aim of this study was to develop and validate a scale that measures the biopsychosocial approach of family physicians to their patients through the Delphi and validation process.

**Setting:**

The scale was developed through the Delphi study and validated by means of significant statistical methods. Pearson’s correlation coefficient, Cronbach’s alpha, the intracorrelation coefficient, the Spearman-Brown coefficient and exploratory factor analysis were applied.

**Participants:**

Five family physicians took part in a brainstorming process and 24 family medicine experts took part in the Delphi study. For the first part of the validation process, there were 31 family medicine trainees in the first group and 32 in the second group. For the last part of the validation process, 164 family physicians completed the scale.

**Result:**

Through the Delphi study, 39 final items covering three areas within the biopsychosocial approach were identified. Construct validity was high, with positive linear correlation and good face validity. The intraclass correlation coefficient for test–retest reliability was 0.862. The Spearman-Brown coefficient was the highest (0.931) on an even and odd division. Factor rotation showed that three factors on 35 items explained 39.5% of variances. The final internal consistency on 35 items was 0.911.

**Conclusion:**

The developed scale measures the biopsychosocial dimension of family physicians’ work with high Cronbach’s alpha measures and good validity.

**What is already known on this topic**

- The biopsychosocial perspective is particularly important at the primary healthcare level, as it enables us to recognise and address all the different needs of patients accordingly. The research was focused on developing and validating the scale for measuring the biopsychosocial dimension of family physicians’ work, since no such scale is yet available.

**What this study adds**

- The design, methods and analysis were appropriate for developing a good and reliable scale. The developed scale is suitable for measuring whether the work of family physicians is biopsychosocially oriented towards their patients.

**How this study might affect research, practice and/or policy:**

- The added value lies in the fact that the scale has good potential for use in the research field, not only with family physicians (where it has already been used) but also with mental health workers at the primary healthcare level.

## Introduction

Engel was the first to introduce the biopsychosocial (BPS) model to the world in 1977. It takes into account biological, psychological and social factors, and their complex interactions, in order to understand health, illness and healthcare delivery.^1^ The BPS approach is important at the primary healthcare level, and the World Organisation of Family Doctors Europe has incorporated it into one of the core competencies of family medicine.^2^ The challenge in primary care is to decide whether a physical complaint represents a physical illness or is a somatic manifestation of a psychological problem.^3^ Health is also influenced by social determinants of health and the conditions in which people live, exacerbated by the distribution of money, power and resources. Health-related behaviours, socioeconomic factors and environmental factors influence health outcomes.^4^ The BPS approach, which considers disease to be the result of organic, human and environmental factors, is closely connected to patient-centred care, which is a key element in ensuring high-quality care.^5^ Despite this, there are still healthcare systems that focus exclusively on physical care and fail to provide mental healthcare to their populations.^6^ Family physicians and their teams take care of those who suffer from the whole spectrum of acute and long-term medical conditions, including mental illness.^7^ With its longitudinal family approach, family medicine can usually recognise mental health issues in their initial phases. To recognise mental health issues and to further collaborate inter-professionally, reduces the treatment gap in mental health.^8^ This is important because mental disorders are prevalent in all countries and are connected to poor quality of life, increased mortality, and high social and economic costs.^6^ Mental illness also complicates other medical conditions, making them more challenging and more expensive to manage, which makes mental health an important issue for primary care physicians as well.

Needs assessment is the systematic evaluation of a person’s needs, conducted in order to plan treatment that can meet identified needs and completed as an interview during a patient’s visit. People do not suffer in terms of isolated organs, but as a whole.^9^ They, therefore, have different needs. It is important for the physician to recognise psychosocial issues, especially in patients with chronic disease, in order to build a good patient–physician relationship and optimise treatment^10^ and address the needs accordingly.

First, before adequate recognition of the patient’s health needs, it is important to evaluate whether family physicians are able and willing to address the broad scope of BPS health needs. As the level of BPS approach has not been measured,^11^ the aim of this study was to develop and validate a scale that measure the BPS approach of family physicians to their patients.

## Methods

**Figure 1:**
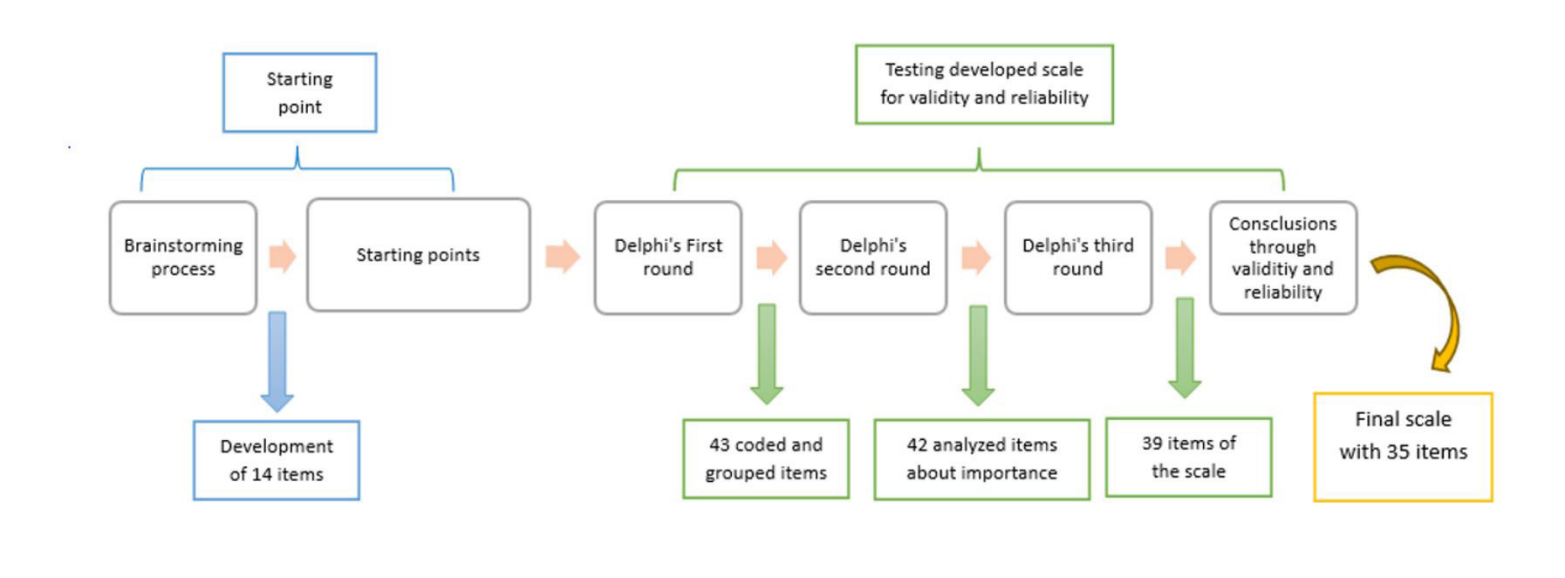
Complete process of development and validation.

The complete process of development and validation, from validity to reliability, is seen (figure 1) and described below.

### Research design

After the literature review,^11^ a qualitative and quantitative study methodology was used in the process of developing the scale. The qualitative part was carried out through a Delphi study process with family physicians and in accordance with research recommendations.^12^ The quantitative part, determining validity and reliability,^13^ as in other studies,^14^ was carried out with the help of family medicine trainees and family physicians through a cross-sectional study. Testing of the scale was also carried out through one part of the cross-sectional study.

### Participants

Different steps of the study involved different participants. Five family physicians, who were also professors of family medicine with more than 20 years of work experience, participated in a brainstorming process that was a prephase of the Delphi study. Thirty-nine family medicine experts were invited to participate in the Delphi study. Invitations were was sent to all family physicians working as mentors for young trainees at the University of Ljubljana’s Faculty of Medicine, but who are situated throughout Slovenia (and therefore cover city and village settings). For one part of validity study, two groups, one comprising 37 and the other 36 family medicine trainees, were invited to complete the scale. All students who were, at that time, part of the educational process at the family medicine department were invited to take part. Finally, 255 family physicians were invited to complete the scale as part of the cross-sectional study. We invited the same sample as the Qualicopc study, since an additional purpose of the validated scale was to measure outcomes on the patients’ side (which is part of another article).

### Development of the scale

#### Delphi process

The scale was developed through several phases. The literature review^11^ and the brainstorming phase, including several starting points for each part of the BPS model, marked the beginning of the Delphi process. The brainstorming process comprised three starting points for each of every part of biopsychosocial part of the model. On the base of the brainstorming starting points within the first Delphi round, additional statements that covered the BPS dimension of family physicians’ work were acquired and consolidated for the second round. In the second Delphi round, participants used a 5-point Likert scale (1—not important at all, 5—very important) to determine whether the item was important for measuring the biopsychosociality of the family physicians’ approach. The goal was to achieve a consensus of at least 75%.^15^ In the third round, the participants received their answers from the second round and the group median, and were asked to answer again (the same or differently) and assess those items that did not achieve agreement about importance or a consensus level. The final scale was designed according to the arithmetic mean. The items were moderated in such a way that they could be answered on a 5-point Likert scale to the question whether the physicians used this type of approach during their everyday work (1—never, 2—rarely, 3—sometimes, 4—often and 5—always).

#### Validity

Content validity was shown through the Delphi process. First, various participants made it possible to identify relevant topics covering all aspects of the biopsychosocially oriented physician’s work. Second, relevance assessment of items was to be included in this scale. Using a 5-point Likert scale, participants stated whether the item was important for measuring the biopsychosociality of the family physicians’ approach with a consensus level of at least 75%. The second step in the development of the scale was to assess its comprehensibility and face validity. Face validity was assessed through an understanding of the questions in the BPS scale. Family medicine trainees were asked to answer whether those items were understandable (1—impossible to understand, 5—totally understandable). Concurrent validity was carried out through a comparison between the newly developed questions and the questions that were already known and were asking the same thing. Predictive validity is not part of this article.

#### Reliability

A reliability study with the second group of family medicine trainees was carried out in two parts to determine test–retest reliability. Family medicine trainees were a group of trainees in family medicine who were currently in the educational process at the family medicine department.

Internal consistency, as a measure of equivalence, was shown through Cronbach’s alpha. The important part of reliability was carried out through factor analysis. Cronbach’s alpha for factors was assessed (with two additional recoded variables, because of their negative values on factors) and the statements with its lowest alphas were eliminated.

The final step was carried out through a cross-sectional study with family physicians that answered the scale with a 5-point Likert scale in order to get the final reliable and validated scale (statistical data for final scale is available in the table 3, where in final scale there are items without blue painted ones).

#### Statistical analysis

The Delphi analysis was conducted using the Atlas.ti programme by coding and grouping the statements. Concurrent validity was shown through Pearson’s correlation coefficient. Two methods were used to measure reliability: test-retest and split halves. Cronbach’s alpha was used as an important indicator to show quality and internal consistency. Exploratory factor analysis using the principal components analysis (PCA) method with promax rotation was applied. Through PCA with the help of Ward’s dendogram, the number of factor groups was seen. The rotation on recoded factors showed the percentage of the explained variances and named those three factors. The SPSS Statistics V.22 program was used to conduct quantitative measurements.

For the Delphi process and for one part of the validation process, there were agreements on cooperation.

## Results

### Demographic characteristics of the samples

In the Delphi study, 24 family medicine experts participated (from the 39 invited), 22 participants responded in the second round and 21 out of 24 participated in the third round. The majority were women. Most of the participants lived and worked in urban environments in Slovenia and ranged in age 33–62. The comprehensibility of the questions was assessed by 31 (out of 36) family medicine trainees. Family medicine trainees were mostly women, aged between 28 and 56. Finally, a second group of family medicine trainees participated in a research study, answering the scale in two separated sets of time. The first round was answered by 32 and the second round by 25 family medicine trainees (out of 37). They were mostly women, living in an urban environment but working in rural settings. For the final validation, 164 family medical doctors participated within a cross-sectional research (out of 255 invited). The majority were women (table 1).

**Table 1:** Details of participants in each step

Participant characteristics		Delphi study	Family medicine trainees first group	Family medicine trainees second group (first/second round)	Family physicians
All variables	n	n=24	n=31	n=32/25	n=164
Age	Mean±SD	48.4±8.6	34.4±8.1	29.8±2.0/29.6±2.0	51.8±7.4
Gender n (%)	Female	14 (64.0)	17 (74.0)	29 (90.6)/21 (84.0)	114 (70.8)
Living environment n (%)	City	14 (64.0)	/	20 (62.5)/13 (52.0)	106 (66.7)
Working environment n (%)	City	14 (64.0)	/	14 (43.8)/12 (48.0)	87 (56.5)
Response rate %	First/second/third round	61.5/91.7/87.5	86.1	86.5/78.1	64.3

### Delphi study

The outcome of the prestep of the Delphi study was 14 items (online supplemental table 2): 5 items for biomedical, 5 for psychological and 4 items for the social part of the model. The first Delphi round produced 43 generated items (9 for the biomedical part, 17 for the psychological part and 17 for the social part) from the 230 initial coded and grouped items. The result of the second round (online supplemental table 3) was the elimination of one item because the median for this was 1 (most participants agreed that it was not important at all). Nine attitudes that did not achieve agreement in the second round were sent to the third round (online supplemental table 4). Three items that still failed to achieve a consensus on their importance (two items with median 3) or did not achieve the consensus level (71.4%) were excluded. This is where the Delphi study ended. The completed Delphi study therefore gave us 39 final items covering three different but interconnected areas: biomedical, psychological and social. Two negatively stated items had an inverted scale, so a recoded item was applied where necessary.

10.1136/fmch-2021-001407.supp1 Supplementary data

### Validity

All the average answers of the questions were higher than 4 (from 4.2 to 4.8). One question (P9) had already been stated negatively, while another (P10) was stated positively here; based on their comments, it was changed to negative for future research. There were three questions (B2, S3, P5) that were positively significantly important and connected with already validated questions. Construct validity showed that there was a positive linear correlation between all three dimensions. All three dimensions had average values of between 3.8 and 4.0 (from 3.3 to 4.4). The highest correlation coefficient was between the social and psychological dimensions (r=0.675; p<0.001), the results of correlation between the psychological and biomedical dimensions were somewhere in between (r=0.352; p<0.001), and the correlation between the social and biomedical dimensions was lowest (r=0.175; p<0.05).

### Reliability

The intraclass correlation coefficient (ICC) for average measures (for test–retest reliability) was 0.862 (ICC 0.778–0.927). There was also high ICC on the social (ICC=0.809) and psychological dimensions (ICC=0.758), but low on the biomedical dimension (ICC=0.380). Moreover, the second certification of reliability, that of split halves, showed that the Spearman-Brown coefficient was high on both samples: family medicine trainees and family medical doctors. The highest coefficient was on even and odd division of the family medical doctors sample (Spearman-Brown=0.931). Cronbach was good for the whole scale, with 39 items (2 of them recoded), but was lower in the sample of family medicine trainees (0.881) than in the sample of family medical doctors (0.893). Factor rotation showed that three factors explained 36.3% of the variances. First factor named: ‘Holistic or social approach’, with 14 statements explaining 24.1% of the variances. Second factor named: ‘Psychological part of family medical doctor’s work’, with 13 statements explaining 6.9% of the variances. Third factor named: ‘Partnership between patient and doctor’, with 12 statements explaining 5.3% of the variances. Cronbach’s alpha was good on the first factor (0.849), and almost good on the second (0.793) and third factors (0.771). In this step, we eliminated four items. The total Cronbach’s alpha on 35 items was 0.911 and also the Keiser-Meyer-Olkin test was higher after the elimination of three items. Three factors with 35 statements explained the higher percentage of variances (39.5%). The final scale for measuring the BPS dimension of the family physician’s work has 35 statements that are given in even-odd arrangement. Those factors present three important parts of the BPS model and those factors are named: holistic approach, partnership (between physician and patient) and the psychological component of a physician’s work. A stratified analysis based on gender and age, as well as a scale on the complete sample, shows (online supplemental table 5) that the average answers range from 3.01 to 4.79 on the whole scale (male from 2.85 to 4.79; female from 2.92 to 4.79; younger than 54 from 2.91 to 4.77, older than 55 from 3.12 to 4.82).

## Discussion

Through a rigorous scale development process, a valid and reliable scale for measuring the level of the BPS approach of family physicians was developed. To the best of our knowledge, this is the first such scale in the scientific literature.^11^

The developed scale for the BPS dimension of the family physician’s (FP) work (BPS Dimension of FP is available in online supplemental material) had good reliability^16^ and validity scores.^17^ According to the statistical results, the final scale was arranged with 35 items in odd and even order. This was shown as the strongest in a highly valued arrangement. The key factors of the scales were holistic approach; partnership between doctor and patient; psychological component of a family physician’s work. Those factors show that there is a BPS dimension in this scale. Pearson’s correlation coefficient also showed a positive linear correlation between developed dimensions of the scale. Between the social and psychological dimensions, there was a strong correlation, between the psychological and biomedical dimensions there was a moderate correlation, and between the social and biomedical dimensions there was a weak correlation. Those three factors are still sufficiently correlated to ensure that the scale is good for measuring the BPS dimension of a family physician’s work. There was also the possibility of correlating the items developed and validated in our scale with some existing international questions that measure some parts of the BPS dimension.^18^ Analysis shows that the distribution has almost perfect symmetry (online supplemental figure 1). This is good because the floor effect is related to skewness to the right, while the ceiling is the opposite.

Our study put forward three factors which together measure the BPS approach of a family physician to the patients. The first factor measures the holistic approach—whether the physician is familiar with the social background of the patient and takes family background, job situation, psychological framework, etc into consideration when applying clinical knowledge. This was also mentioned in one focus group study, where a doctor said that some patients used sick notes for their condition as a kind of preventive measure.^19^ The second factor is about the psychological part of the family physician’s work–knowledge and skills for knowing the special work of a family physician, which is being aware of the psychological context of an individual’s health, as well as their psychological health. As other studies have shown, BPS framing is centred on how to improve the patient’s situation^19^ and therefore it is more oriented towards the patient’s context. The third factor is the partnership between the patient and the doctor—the individual approach and cooperation with the patient. Patient communication is a critical component of quality of healthcare,^20^ and patient centredness is therefore an important third factor for the further process of measuring whether different levels of biopsychosociality result in different healthcare quality outcomes. Other studies also show that psychosocial factors interfere with health-related quality of life.^21^

A holistic approach or person-oriented care in combination with a patient-centred approach (with shared decision making) is an important part of the BPS framework,^22^ and both are therefore important factors for a tool that aims to measure the level of biopsychosociality.

Nevertheless, this scale is only one part of the whole picture.^23^ Self-assessment of family physicians regarding the style or type of their work is individually assessed and could potentially be subjected to biased assessment, with an awareness of how it is expected to approach the patients. On the other hand, there is also a system that enables (or not) a specific kind of work. Within this BPS perspective of a family physician’s work, there are also patients with their needs, interests and expectations (this connection has been assessed elsewhere^24^). The developed scale is a starting point for research into the BPS model in practice, and enables a path through information and knowledge to understanding.^23^

This developed scale could potentially be used as a research form in the future. One study shows that family physicians with the most experience mostly employ the BPS frame, whereas those with the least experience rely more on the biomedical frame,^19^ which is the next possibility of this scale that could be used in further studies to ascertain whether there is a correlation with additional factors. Comparisons within different countries with similarly organised primary care healthcare could be useful. Use of this scale can be also a good reminder for family physicians that biopsychosocially oriented work that assesses a patient’s needs on all three closely connected levels (biomedical, psychological and social) is the norm. A major strength of our study is the multistage iterative process that was used to develop the scale. With the development of the tool through the inclusion of quantitative and qualitative methods, an all-encompassing scale^23^ has been generated that focuses on personal information (psychological part or mental health), community health (social part) and the provision of healthcare (biomedical part or physical health).

The positive part of the Delphi study methodology is that participants are not associated with each other and therefore cannot influence each other’s opinions, while the limitation of this process is in the seeking of a consensus without discussion among participants. Because the Delphi technique is usually used to obtain views,^15^ there was a need to validate the scale. This problem was solved by statistical analysis. The concurrent validity was partly tested through the connection with only three items, but not through the whole scale. This is because our scale is new in this field. Predictive validity, which shows whether this scale can predict the outcome that the more BPS the physicians’ approach, the more satisfied are the patients, is not shown for the purpose of this article.

## Conclusion

The BPS scale is a reliable, valid and useful self-reported scale for measuring the BPS dimension of family physicians toward their patients. It could be used in different healthcare systems, comparisons between different countries could be carried out, and findings could be connected to the organisation of the healthcare system. This scale could potentially be used to study what else is influencing the level of doctors’ BPS orientation. Further investigations could show whether the level of biopsychosociality influences quality outcomes: is the treatment of the patient better, are the patients more satisfied, etc. Scale could potentially be transferred to other primary healthcare settings, such as community mental healthcare settings.

## Data Availability

Data are available on reasonable request. Some data are available as online supplemental information. Other data are available on reasonable request.
