# Supplementary material for: Development and validation of the scale for measuring biopsychosocial approach of family physicians to their patients
Source: Fam Med Community Health. 2022 May 16;10(2):e001407. doi: 10.1136/fmch-2021-001407 (PMC9114858; doi:10.1136/fmch-2021-001407)
Supplement: Supplementary data [file fmch-2021-001407supp001.pdf]

BPS Dimension of FP

Scale for BioPsichoSocial Dimension of Family Physician' Work

		1–never, 2–rarely, 3–sometimes, 4–very often, 5–always
B3	During the consultation I apply the relevant diagnostics, which include a medical history and clinical examination oriented towards the clinical symptoms of the patient.	1 2 3 4 5
B5	At each consultation I complete the medical records (entering all the issues) and other documents (checking that all results are included).	1 2 3 4 5
B7	Within my work I communicate and cooperate (consultation vie telephone or via e-mail) with clinical specialist (before and/or after referral).	1 2 3 4 5
P1	During the consultation, as well as verbal communication (speech), I use careful listening to the patient, observation, and contact (handshake, looking with understanding), even when a physical check is not urgently required.	1 2 3 4 5
P3	I treat the patient with respect to his personality, personal values and fears, and adapt the consultation accordingly.	1 2 3 4 5
P5	At each consultation, when examining the patient, I ask about the psychological background of the state of their disease and how they feel.	1 2 3 4 5
P11	It is essential for my work to be in a partnership with a patient since I often involve patients in co-decisions.	1 2 3 4 5
P13	For each patient, I know their relationship to health and disease, their experiences, wishes, viewpoints, how they accept their disease and so on.	1 2 3 4 5
P15	The work of the family doctor, compared with other specialists, requires a special relationship between the doctor and the patient and the character of the doctor (which I possess).	1 2 3 4 5
P17	I express clear empathy towards my patients.	1 2 3 4 5
S1	Trust and division of work amongst team members contributes to the successful management of patients.	1 2 3 4 5
S3	I know what the patient's life is like - what is happening at home, what job they have, what their relationships are like, etc.	1 2 3 4 5
S5	With elderly patients, I always check again what their access to health care is.	1 2 3 4 5
S7	In order to save (time and money) I consider the quality of patient care (holistic, taking into account all problems, etc.) at their first and at every subsequent visit.	1 2 3 4 5
S9	I check how the disease affects the patient's working environment, family, etc.	1 2 3 4 5
S11	During the consultation I always consider the patient's personality and emotional intelligence.	1 2 3 4 5

S13	When I see that the patient has problems that are not strictly medical, I direct them to the appropriate place, for example, the social work department. At their next visit, I check whether the situation has been successfully resolved.	1 2 3 4 5
B2	As part of my work I undertake professional training in the clinical field, and I always follow professional guidelines and monitor all new developments.	1 2 3 4 5
B4	I am aware of the limits to my abilities, possibilities and obligations in my treatment of the patient's problems.	1 2 3 4 5
B6	When I prescribe treatment, I know the medicines and check which drugs the patient is already using.	1 2 3 4 5
P2	I am able to read the patient's non-verbal communication and respond differently in view of their emotional reactions.	1 2 3 4 5
P4	For the good treatment of my patients, I believe it is crucial to have good cooperation as well as mutual respect and trust on both sides.	1 2 3 4 5
P6	I am always careful to maintain a professional attitude towards patients.	1 2 3 4 5
P8	I focus my examination on the psychological profile of the patient, as many physical symptoms result from mental pressure and problems.	1 2 3 4 5
P10	Chronic patients require a lot of time, so I often deal with them in less depth.	1 2 3 4 5
P12	With patients I am a counsellor and an advocate for their rights.	1 2 3 4 5
P14	I am patient and tolerant even towards people who think differently, and those of other cultures, languages, orientations, and so on.	1 2 3 4 5
P16	I pay special attention to patients who have poorly differentiated status and/or multimorbidity.	1 2 3 4 5
S2	I consider the patient's social environment in every disease and all their symptoms.	1 2 3 4 5
S4	The quality of life of my patients (discussion and explanation of the course of the disease, expectations, etc.) is more important to me than (merely) the clinical objectives of healing or not healing.	1 2 3 4 5
S6	I know the family history of all my patients - the developmental stage of the family, their ability to look after the patient, who their partner is, who offers them support, the health of their parents and so on.	1 2 3 4 5
S8	I know the work situation and work environment of my patients.	1 2 3 4 5
S10	I am familiar with the social circumstances (financial situation, lifestyle) and social situation of the patient.	1 2 3 4 5
S12	I know the cultural, ethical (how they live, what their work is, which moral principles they follow) and religious background of the patient.	1 2 3 4 5
S14	Part of my doctor's work is focused on the community and I also carry this responsibility (I voluntarily participate in social and health organizations and associations).	1 2 3 4 5

Results of each Delphi Round

Table 2: Results of brainstormin process

MODEL PART	***ITEM***
BIOMEDICAL	Clinical knowledge is the foundation.
	Knowledge of clinical parameters is important.
	Guidelines are important.
	Differential diagnostics is important.
	The correct anamnesis is crucial.
PSYCHOLOGICAL	Empathy is important in the work of family physician.
	Cooperation and partnership between the doctor and the patient are important for quality treatment.
	Respect is necessary for a doctor's successful work.
	Patient-oriented, individual-specific treatment is important.
	Long-term cooperation with the patient is crucial for the good work of a family physician.
SOCIAL	The patient's environment is of great importance for achieving health.
	The doctor must know the patient's family.
	The doctor must take into account the patient's financial environment during treatment.
	The doctor needs to know the patient's working enviroment.

Table 3: Items of the biopsychosocial dimension scale after 2nd round of the Delphi study (averages, deviations, means and consensus level)

	Aritmetic mean/ stand.deviation	Median	Mode	Consesus level (%)
BIOMEDICAL DIMENSION				
Clinical knowledge and skills are the foundation of a family doctor’s work.	4,8 / ±0,501	5	5	95,5
Every disease can be cured if we know the cause.	3,7 / ±0,856	4	4	**66,6**
Family physician must constantly improve professional skills, follow professional guidelines and keep up to date (in their area of competence).	4,7 / ±0,456	5	5	100,0

An appropriate approach to diagnosis is important (anamnesis, targeted examination of the patient) .	4,6 / ±0,498	5	5	100,0
A focused clinical examination that focuses on pathophysiological changes makes the most sense.	3,8 / ±0,928	4	4	81,0
Good collaboration and communication with clinical specialists is important.	4,0 / ±0,617	4	4	81,8
When prescribing medications it is necessary to know their action and to prevent polypharmacy.	4,4 / ±0,581	4	4	95,4
Well-managed medical documentation contributes to the quality of the treatment.	4,5 / ±0,602	5	5	95,3
The doctor must be aware of the limits of his/her possibilities and duties.	4,5 / ±0,598	5	5	95,4
PSYHOLOGICAL DIMENSION				
The management of chronic patients is important.	4,3 / ±0,550	4	4	95,4
Conversation, observation, listening, reviewing, touching are irreplaceable.	4,8 / ±0,429	5	5	100,0
Psychological problems and / or multimorbidity require more medical time, but should always be taken into account, regardless of time wasting.	4,7 / ±0,658	4	4	91,0
A feature of family medicine is the treatment of poorly differentiated conditions.	3,8 / ±0,853	4	4	**63,6**
The holistic approach of the doctor is important (we ask the patient about well-being, psychological background, etc.), as it affects the results of the treatment.	4,6 / ±0,503	5	5	100,0
Strongly individualized treatment tailored to the specific patient is important (knowledge of the patient's personality, his values, fears).	4,6 / ±0,581	5	5	95,5
It is important that the primary care physician expresses high empathy.	4,0 / ±0,690	4	4	77,2
Good cooperation, respect and trust between the doctor and the patient are important for quality treatment.	4,6 / ±0,658	5	5	90,9
The physician must be able to communicate well (and also read the patient's non-verbal communication), and respond appropriately to the patient's emotional reactions.	4,7 / ±0,489	5	5	100,0

The doctor must also be patient and tolerant of dissenters (different culture, language, orientation).	4,1 / ±0,710	4	4	81,8
It is important to know the patient's attitude towards health and illness (patient's experiences, wishes, attitudes, acceptance of illness…).	4,2 / ±0,612	4	4	90,9
Partnership, patient involvement in treatment and co-decision are important.	4,3 / ±0,550	4	4	95,4
Professional attitude is important in the work of a family physician.	4,4 / ±0,590	4	4	95,5
It is important that the doctor takes good care also about its own psychophysical condition.	4,4 / ±0,666	4,5	5	90,9
The physician should be aware that many of the physical symptoms of the disease stem from mental distress or problems.	4,4 / ±0,734	4,5	5	95,5
In the work of a doctor, his personality is also important.	4,1 / ±0,750	4	4	77,3
The physician is the patient's advisor and advocate.	3,9 / ±0,868	4	4	**68,2**
SOCIAL DIMENSION<br>Family physician must know the patient's family (developmental stage, relationships and ability to care for the patient within the family): newborn, adolescents, dying / ill parents, etc.	4,1 / ±0,610	4	4	86,3
Successful treatment of patients requires trust and division of labor among team members.	4,6 / ±0,598	5	5	95,2
The patient must change doctor every now and then.	1,7 / ±0,883	**1**	**1**	**0,0**
A good treatment at the primary level can save a lot of public money.	4,1 / ±1,065	4	4	81,8
Family physician must know the patient's existential aspects (events at home, work, relationships).	4,1 / ±0,56	4	4	90,9
Quality of life is more important than only clinical goals.	4,2 / ±0,853	4	5	**72,8**
The treatment must take into account the patient and his social environment.	4,4 / ±0,581	4	4	95,4
The doctor should take into account the patient's financial environment during the treatment.	3,5 / ±0,740	3	3	**45,5**
The doctor needs to know the patient's working environment.	4,0 / ±0,690	4	4	86,4

The doctor must know the cultural / ethical / religious background of the patient.	3,9 / ±0,539	4	4	80,9
The doctor must know the social (finances, lifestyle, social position) position of the patient.	3,8 / ±0,602	4	4	**71,4**
It is important to check the condition of the elderly and their access to health care.	4,1 / ±0,539	4	4	90,4
Family physician is part of the community and his responsible work must be community-oriented.	3,9 / ±0,640	4	4	**72,7**
The doctor must know how to help the patients or how to direct them to the right address for problems that are not narrowly medical.	3,8 / ±0,733	4	4	**72,7**
The patient's personality and emotional intelligence affect the disease / treatment.	3,9 / ±0,653	4	4	86,3
The illness affects the patient's social life (work environment, family).	4,0 / ±0,436	4	4	90,9
Relatives cannot influence on doctor’s decisions.	3,5 / ±0,671	3	3	**45,4**

Table 4: Items of the biopsychosocial dimension scale after 3rd round of the Delphi study (median, mode and consesus level)

	Consensus level 2nd round (%)	Median 2nd round	Median 3rd round	Consensus level 3rd round (%)
Every disease can be cured if we know the cause.	66,6	4	4	**71,4**
A characteristic feature of family medicine is the treatment of poorly differentiated conditions.	63,6	4	4	90,0
Family physician is the patient’s advisor and advocate.	68,2	4	4	95,3
Quality of life is more important than only clinical goals.	72,8	4	4	75,0
The doctor should take into account the patient's financial environment during the treatment.	45,5	3	3	**47,7**

The doctor must know the social (finances, lifestyle, social status) position of the patient.	71,4	4	4	80,0
Family physician is part of the community and his responsible work must be community-oriented.	72,7	4	4	80,9
The doctor must know how to help the patients or how to direct them to the right address for problems that are not narrowly medical.	72,7	4	4	81,0
Relatives cannot influence on doctor's decisions.	45,4	3	3	**30,0**

Validation

Picture 1: Histogram showing kurtosis and skewness for floor and ceiling effect

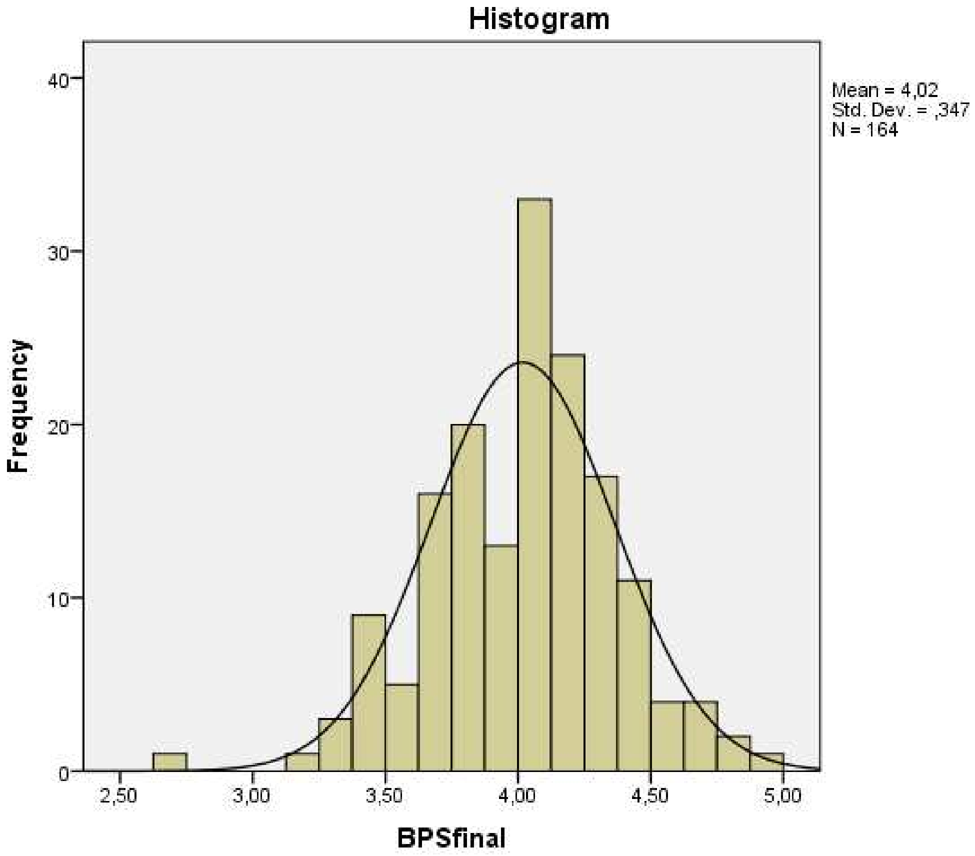

Table 5: Mean and standard deviation and stratified analysis based on gender and age

	Whole sample		Gender				Age			
Scale			*Male*		*Female*		*Younger than 54*		*Older than 55*	
Items	Mean	Std. Deviation	Mean	Std. Deviation	Mean	Std. Deviation	Mean	Std. Deviation	Mean	Std. Deviation
B3	**4,79**	0,410	**4,79**	0,414	**4,79**	0,411	**4,77**	0,424	**4,82**	0,391
B5	4,57	0,600	4,36	0,705	4,64	0,534	4,58	0,556	4,52	0,664
B7	3,20	0,930	**2,85**	0,908	3,33	0,889	3,22	0,943	**3,12**	0,857
P1	4,19	0,700	4,09	0,830	4,22	0,635	4,19	0,621	4,15	0,795
P3	4,30	0,680	4,21	0,858	4,33	0,605	4,34	0,630	4,22	0,760
P5	3,68	0,710	3,49	0,804	3,76	0,656	3,69	0,701	3,65	0,717
P11	4,12	0,720	4,13	0,694	4,11	0,736	4,07	0,688	4,16	0,766
P13	3,65	0,650	3,71	0,727	3,63	0,628	3,63	0,669	3,67	0,644
P15	4,38	0,650	4,44	0,586	4,36	0,680	4,36	0,651	4,41	0,660
P17	4,09	0,700	3,96	0,767	4,15	0,660	4,13	0,572	4,03	0,842
S1	4,71	0,500	4,75	0,438	4,68	0,521	4,68	0,490	4,75	0,507
S3	3,56	0,690	3,49	0,757	3,58	0,664	3,49	0,666	3,64	0,721
S5	3,83	0,770	3,73	0,809	3,88	0,754	3,74	0,761	3,95	0,765
S7	3,83	0,760	3,91	0,812	3,79	0,753	3,82	0,768	3,83	0,773
S9	3,78	0,660	3,78	0,664	3,78	0,667	3,76	0,628	3,79	0,722
S11	4,18	0,650	4,20	0,719	4,18	0,633	4,18	0,598	4,19	0,737
S13	3,91	0,750	3,83	0,825	3,95	0,721	3,80	0,749	4,08	0,725
B2	4,14	0,570	4,11	0,667	4,17	0,531	4,17	0,474	4,12	0,696
B4	4,55	0,540	4,57	0,542	4,55	0,534	4,53	0,543	4,58	0,527
B6	4,46	0,560	4,38	0,644	4,50	0,520	4,47	0,522	4,48	0,615
P2	4,13	0,670	4,19	0,680	4,10	0,665	4,08	0,675	4,17	0,651
P4	4,74	0,470	4,74	0,441	4,75	0,476	4,73	0,492	4,75	0,434
P6	4,60	0,550	4,51	0,585	4,64	0,534	4,58	0,536	4,63	0,575
P8	3,75	0,660	3,78	0,704	3,73	0,642	3,73	0,675	3,75	0,642
P10r	3,47	0,920	3,22	1,085	3,57	0,830	3,53	0,770	3,39	1,107
P12	4,07	0,710	4,02	0,690	4,07	0,713	4,00	0,668	4,13	0,745
P14	4,38	0,580	4,51	0,549	4,32	0,587	4,37	0,584	4,38	0,577
P16	4,28	0,610	4,29	0,727	4,26	0,565	4,23	0,627	4,34	0,597
S2	3,75	0,690	3,69	0,701	3,79	0,687	3,78	0,671	3,71	0,728
S4	3,83	0,730	3,84	0,796	3,82	0,711	3,84	0,748	3,78	0,701
S6	3,61	0,760	3,51	0,869	3,66	0,714	3,51	0,756	3,78	0,745
S8	3,80	0,650	3,76	0,705	3,82	0,633	3,79	0,631	3,83	0,685
S10	3,57	0,760	3,65	0,822	3,54	0,734	3,53	0,739	3,63	0,789
S12	3,61	0,800	3,80	0,806	3,54	0,793	3,56	0,737	3,70	0,891
S14	**3,01**	1,170	3,24	1,214	**2,92**	1,148	**2,91**	1,115	3,17	1,238
BPS final	4,02	0,350	3,99	0,404	4,03	0,326	3,99	0,329	4,04	0,371
